# The *NFIA::CBFA2T3* identifies a molecularly defined subgroup of acute erythroid leukemia/erythroid sarcoma

**DOI:** 10.3389/fonc.2026.1809156

**Published:** 2026-05-04

**Authors:** Marta Brunetti, Kristin Andersen, Geir Erland Tjønnfjord, Bernward Zeller, Signe Spetalen, Monica Cheng Munthe-Kaas, Liv Toril Nygård Osnes, Tina Treu Os, Francesca Micci

**Affiliations:** 1 Section for Cancer Cytogenetics, Department of Pathology, Oslo University Hospital, Oslo, Norway; 2 Department of Hematology, Oslo University Hospital, Oslo, Norway; 3 Institute of Clinical Medicine, Faculty of Medicine, University of Oslo, Oslo, Norway; 4 Department of Pediatrics, Oslo University Hospital, Oslo, Norway; 5 Department of Pathology, Oslo University Hospital, Oslo, Norway; 6 Department of Immunology, Oslo University Hospital, Oslo, Norway

**Keywords:** erythroid leukemia, erythroid sarcoma, fusion gene, *NFIA::CBFA2T3*, pediatric leukemia, t (1,16)(p31;q24)

## Abstract

**Introduction:**

The World Health Organization classification of tumors of hematopoietic and lymphoid tissues recognizes acute erythroid leukemia as a distinct entity under myeloid neoplasms. Erythroblastic sarcoma, also known as extramedullary erythroid sarcoma, is defined as a rare extramedullary tumor composed of erythroid precursors, typically occurring outside the bone marrow. While both entities involve immature erythroid precursors and share similar histology, acute erythroid leukemia is a systemic marrow-based leukemia, whereas erythroblastic sarcoma is a localized mass-forming lesion. Since these entities can overlap morphologically with other clinically distinct myeloid malignancies and myelodysplastic syndrome, it is fundamental to characterize them genetically.

**Materials and methods:**

We report a rare and diagnostic challenging case of a 2-year-old boy with erythroid sarcoma characterized by extensive extramedullary involvement, including mediastinum, gastrointestinal tract, pleura/peritoneum, bilateral kidneys, and lymph nodes.

**Results and discussion:**

The patient achieved a sustained complete remission following standard acute myeloid leukemia-like therapy with consolidative allogenic stem cell transplant. An *NFIA::CBFA2T3* chimeric fusion was found in the tumor genome. Since such a fusion transcript has been identified only in cases of pediatric erythroleukemia/erythroid sarcomas, we propose that it may characterize a molecularly defined subgroup of leukemias. The patient is in full remission with no signs of graft-versus-host disease twenty-eight months after the transplant.

## 1 Introduction

Acute erythroid leukemia (AEL) is a rare and aggressive subtype of acute myeloid leukemia (AML). There is an overall erythroid predominance (>80% of the marrow cellularity), which includes immature erythroid precursors (≥30% proerythroblasts) in the bone marrow, with no significant myeloblastic component (1). Extramedullary sarcomatous presentations are reported, though extremely rare. According to the World Health Organization (WHO) classification of tumors of hematopoietic and lymphoid tissues, they are termed myeloid sarcoma with erythroid differentiation, but in the literature, they are also reported as erythroid sarcoma (ES) and erythroblastic sarcoma (2, 3). ES can be associated with acute myeloid leukemia (AML) or other myeloid neoplasms (4), it may also occur as an isolated (*de novo*) case (5) or develop from a pre-existing myelodysplastic syndrome (MDS) or myeloproliferative neoplasm (MPN) (3). AEL/ES have been observed in ovaries (6), lymph nodes (2), orbits (7–9), central nervous system (5, 10, 11), abdomen (12, 13), and parathyroid glands (14). The diagnosis can be challenging due to the nonspecific cytomorphology, lack of expression for CD34 and myeloperoxidase (MPO), and weak or no expression for erythroid-specific markers. CD45 expression is variable but often dimly positive. Most patients are elderly, but AEL/ES may affect children (1) or may occur as a congenital disease (15).

AEL/ES shares cytogenetic and molecular abnormalities with AML (16), including *RUNX1T1* fusions (12). In pediatric cases of AEL, a recurrent chromosomal aberration,t(1;16)(p13;q24), has been identified, leading to the *NFIA::CBFA2T3* fusion transcript (5, 10, 13, 14, 17, 18). Variants or analogs of this rearrangement have also been reported, e.g., t(1;8)(p31;q21) with a *NFIA::RUNX1T1* fusion (12, 13) and t(11;20)(p11;q11) with a *ZMYND8::RELA* fusion (13, 19).

We report a rare and diagnostically challenging case of a 2-year-old boy with AEL/ES showing a *NFIA::CBFA2T3* chimeric fusion in the tumor genome. This provides further information that such a transcript is not only recurrent but may also identifies a distinct, genetically defined, entity of leukemias.

## 2 Materials and methods

### 2.1 Ethics statement

The study was approved by the Regional Committee for Medical and Health Research Ethics South East Norway, and written informed consent was obtained from the patient’s parents to publish the case details.

### 2.2 Patient

A 2.5-year-old boy presented with a 5–6 weeks history of constipation and a 6-day history of vomiting. Upon admission, the patient exhibited abdominal distention, severe dehydration, and renal failure. Laboratory findings revealed metabolic acidosis, elevated serum creatinine levels, but normal hematological values and hepatic transaminases. An abdominal computed tomography (CT) imaging examination demonstrated thickening of the colonic wall in the sigmoid colon and rectum, along with edematous kidneys and bilateral pleural effusions. Subsequent investigations were performed with repeated abdominal ultrasounds and magnetic resonance imaging (MRI) of the abdomen, brain, and spine, as well as gastroscopy and colonoscopy including biopsies were negative. Due to clinical deterioration, a follow-up abdominal MRI and bilateral bone marrow biopsies and aspirates were performed on day 16 post admission. The MRI identified widespread tumor masses involving the abdomen, mediastinum, and collum with infiltration into kidneys, the peritoneum, and pleura. However, none of these were considered accessible for biopsy. The bone marrow investigations were initially reported as normal. Over the following 48 hours, however, the patient’s condition deteriorated further as he developed tachycardia, tachypnea, severe abdominal pain, massive pleural effusions and ascites, hypertension, and generalized edema. By day 17 post admission, pleural and ascites drainage was required, and during the same procedure a lymph node biopsy was taken. During this procedure, nodular infiltration throughout the pleura and peritoneum was observed. Flow cytometric analysis of the pleural effusion revealed a malignant process with the cells testing positive for CD34. Blood tumor markers (Neuron-Specific Enolase, Alpha-Fetoprotein and Human Chorionic Gonadotropin) and urinary catecholamines (Vanillylmandelic Acid and Homovanillic Acid) were normal. Given the patient’s significant clinical deterioration due to respiratory failure, empiric leukemic therapy was initiated on day 18 post admission, despite the absence of a definitive diagnosis. A regimen consisting of etoposide and prednisolone was chosen to cover the main forms of leukemia. One day after etoposide treatment was commenced, the final results of laboratory analyses of cells from the pleural effusion and a lymph node biopsy from his neck raised the suspicion of an erythroid sarcoma. Flow cytometry of the pleural effusion revealed a cell population with the following phenotype: CD45-, CD117+ strong, CD71+ strong, CD36+ strong, CD235a+ strong, CD105-, CD13-, CD33-, CD43+, CD56+weak, and cy MPO-, whereas all lymphoid markers were negative; this suggested the possibility of an erythroid sarcoma (**Supplementary Figure 1**). Reanalysis of the bone marrow aspirate by flow cytometry concluded that 0.08% of the blasts showed the same phenotype as was found in pleural effusion cells. This finding was below the detection limit of biopsy-based assessment, and so the WHO diagnostic criteria for a diagnosis of acute erythroid leukemia were not fulfilled in the bone marrow biopsies. On obtaining these preliminary results, the patient was initiated on AML-like therapy as per the NOPHO AML 2012 protocol with significant clinical improvement (20). He entered a complete remission after the second course of therapy and received consolidative allogenic stem cell transplant following the third course, five months after the patient’s initial admission to the hospital. Twenty-eight months after the transplant, he remains in full remission, with no signs of graft-versus-host disease (GvHD) or other sequelae.

### 2.3 Morphologic evaluation and immunohistochemistry

**Figure 1:**
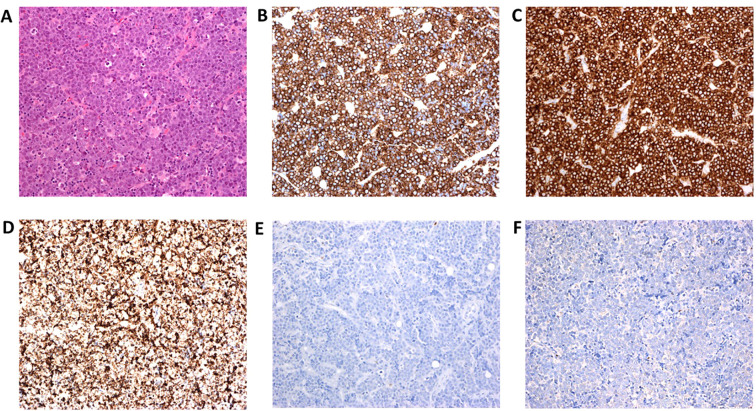
Microscopic examination of the lymph node. **(A)** Hematoxylin and eosin staining (X 200) shows diffuse infiltration of cells with amphophilic cytoplasm and medium-to-large nuclei with pale chromatin and several distinct nucleoli. The cells express CD43 **(B)**, CD117 **(C)**, and E-Cadherin **(D)**. MPO **(E)**, and CD61 **(F)** are negative.

Hematoxylin and eosin–stained sections of the lymph node demonstrated diffuse infiltration by uniform cells, characterized by a distinct rim of amphophilic cytoplasm and medium-sized to large nuclei with finely dispersed chromatin and nucleoli. Erythroid lineage was ascertained by the following phenotype: E-Cadherin+, CD43+, CD117+, TdT-, CD30-, CD34-, CD45-, CD61-, MPO-, CD3-, CD7-, Pax5- (**Figure 1**). The diagnosis was compatible with a myeloid sarcoma, differentiated as an erythroid sarcoma.

The bone marrow biopsy showed a normal distribution of the three hematopoietic cell lineages, with no significant left shift in erythropoiesis. The WHO diagnostic criteria for acute erythroid leukemia were not fulfilled, as erythroid predominance (typically ≥80%), including ≥30% proerythroblasts, was not observed.

### 2.4 G-banding and fluorescence *in situ* hybridization analyses

Samples from bone marrow, lymph nodes, and pleural fluid were sent for cytogenetic investigations. Cells from all samples were short-term cultured and harvested. Chromosome preparations were G-banded using Leishman’s stain (Sigma-Aldrich, St. Louis, MO, USA), and karyotyped according to the guidelines of the International System for Cytogenomic Nomenclature (21). Routine FISH investigations for AML and ALL cases were performed according to standard protocol with commercially available probes (Cytocell, Oxford Gene Technology, Begbroke, Oxfordshire, UK). Furthermore, probes derived from bacterial artificial chromosomes (BAC) were used to identify the involvement of *NFIA* and *CBFA2T3* genes. The commercially available clones RP11-436K8, RP11-32I17, RP11-1K15, and RP11-119G24 (BACPAC Genomics, Emeryville, CA, USA), mapping on 1p31 and covering the *NFIA* gene, were labelled with green. In contrast, the clones CTD-3010L24, CTD-2555A7, and RP11-1122C1 (Invitrogen, Thermo Fisher Scientific, Carlsbad, CA, USA), mapping on 16q24 and covering the *CBFA2T3*, were labelled with red.

### 2.5 DNA and RNA extraction

DNA was extracted from bone marrow cells using the Maxwell 16 Instrument System and purified with the Maxwell 16 Cell DNA Purification Kit (Promega, Madison, WI, USA) or by using EZ1 Advanced XL (QIAGEN, Hilden, Germany) according to the manufacturer’s recommendations. The concentration was measured using a QIAxel microfluidic UV/VIS spectrophotometer (QIAGEN) and a Quantus fluorometer (Promega). Total RNA was extracted using the miRNeasy Mini Kit and QIAcube automated purification system according to the manufacturer’s instructions (QIAGEN). The concentration was then measured using the QIAxpert microfluidic UV/VIS spectrophotometer (QIAGEN). RNA quality was assessed using an Agilent RNA 6000 Nano total kit on an Agilent 2100 Bioanalyzer (Agilent Technologies, Santa Clara, CA, USA).

### 2.6 Array comparative genomic hybridization

aCGH was performed using CytoSure array products (Oxford Gene Technology, Begbroke, Oxfordshire, UK) following the company’s protocols. The slides (CytoSure Cancer +SNP array) were scanned with the Agilent Sure Scan Dx microarray scanner using Agilent Feature Extraction Software (version 12.1.1.1). Data were analyzed using Agilent Feature Extraction Software (Agilent Technologies; version 10.7.3.1) and CytoSure Interpret Software (Oxford Gene Technology; version 4.9.40). Annotations were based on the human reference sequence GRCh37/hg19.

### 2.7 Molecular investigations

Gene variants and presence of known fusion transcripts were detected using the VARIANTPlex relevant to myeloid malignancies (Archer DX, Boulder, 2477 55th St #202, United States), the HemaVision 28Q RT-qPCR kit (DNA Diagnostic, Risskov, Denmark), and the FUSIONPlex, Pan-Heme panel (Archer DX) were used. The products were then sequenced on the NextSeq 2000 (Illumina, San Diego, CA, United States). Data analyses were performed using the companies’ recommended software, specifically Archer Analysis 6.2.7 or Variant Studio 3.0, respectively. Annotations were based on the human reference sequence GRCh37/hg19 and pathogenicity was determined by using relevant databases: Molecular Tumor Board Portal (MTB) Portal (Karolinska Institute), Human Somatic Mutation Database (HSMD; QIAGEN), ClinVar (National Institute of Health), Catalogue of Somatic Mutations in Cancer (COSMIC; Sanger Institute) and the WHO classification.

### 2.8 RNA sequencing

Two hundred nanograms (ng) of total RNA was sent for high-throughput paired-end RNA sequencing to the Genomics Core Facility, Norwegian Radium Hospital, Oslo University Hospital (https://oslo.genomics.no/). The software FusionCatcher was used to find fusion transcripts (22).

### 2.9 Reverse transcription polymerase chain reaction and Sanger sequencing analyses

cDNA was synthesized from 200 ng of total RNA using the iScript Advanced cDNA Synthesis Kit for RT-qPCR according to the manufacturer’s instructions (Bio-Rad, Hercules, CA, USA). cDNA corresponding to 10 ng of total RNA was used as template in subsequent PCR assays using the following primer combination: NFIA-1080F/CBFA2T2-964R and CD74-699F/CAMK2A-280R. Primers used for PCR reactions are listed in **Table 1**. The first PCR amplifications were run on a C-1000 Thermal cycler (Bio-Rad) with an initial denaturation at 94°C for 30 sec, followed by 35 cycles at 98°C for 7 sec and at 58°C for 30 sec, and a final extension at 72°C for 5 min. The amplified fragments were purified using the MinElute PCR Purification Kit (QIAGEN) and subsequently sequenced using an Applied Biosystems SeqStudio Genetic Analyzer system with the BigDye Direct Cycle Sequencing Kit according to the company’s recommendations (ThermoFisher Scientific, Waltham, MA, USA). The primer combinations M13-NFIA-1080-FW/M13-CBFA2T3-799-REV and M13-CD74-719F/M13-CAMK2A-265R were used for Sanger sequencing. The Basic Local Alignment Search Tool software (BLAST; https://blast.ncbi.nlm.nih.gov/Blast.cgi) was used for computer analysis of sequence data (23). The BLAT alignment tool and the human genome browser at the University of California, Santa Cruz (UCSC) were also used to map the sequences on the Human GRCh37/hg19 assembly (BLAT; http://genome.ucsc.edu/cgi-bin/hgBlat) (24).

**Table 1:** Primers used for PCR analysis.

Designation	Sequence	Position	Gene	Accession no.
NFIA-1080F	TGAAATGGACAGTCCTGGTGAGG	1080-1102	*NFIA*	NM_001134673.4
CBFA2T2-964R	CAATCTCTGGGGAGATGTCGCTG	964-986	*CBFA2T3*	NM_005187.6
M13-NFIA-1080-FW	**TGTAAAACGACGGCCAGTTGAAATGGACAGTCCTGGTGAGG**	1080-1102	*NFIA*	NM_001134673.4
M13-CBFA2T3-799R	**CAGGAAACAGCTATGACCCACCATGATGG**CTGTTGGTGAG	799-821	*CBFA2T3*	NM_005187.6
CD74-699F	GTCTTTGAGAGCTGGATGCACCA	699-721	*CD74*	NM_001025159.3
CAMK2A-280R	CTGGCTGACAGCTTCTTTGTGTTG	280-303	*CAMK2A*	NM_015981.4
M13-CD74-719F	**TGTAAAACGACGGCCAGT**CCATTGGCTCCTGTTTGAATGA	719-741	*CD74*	NM_001025159.3
M13-CAMK2A-265R	**CAGGAAACAGCTATGACCTTGTGTTGATGA**TCTTGGGCAAGCA	265-287	*CAMK2A*	NM_015981.4

In bold: the sequences of the forward and reverse M13-tailed primers.

## 3 Results

**Figure 2:**
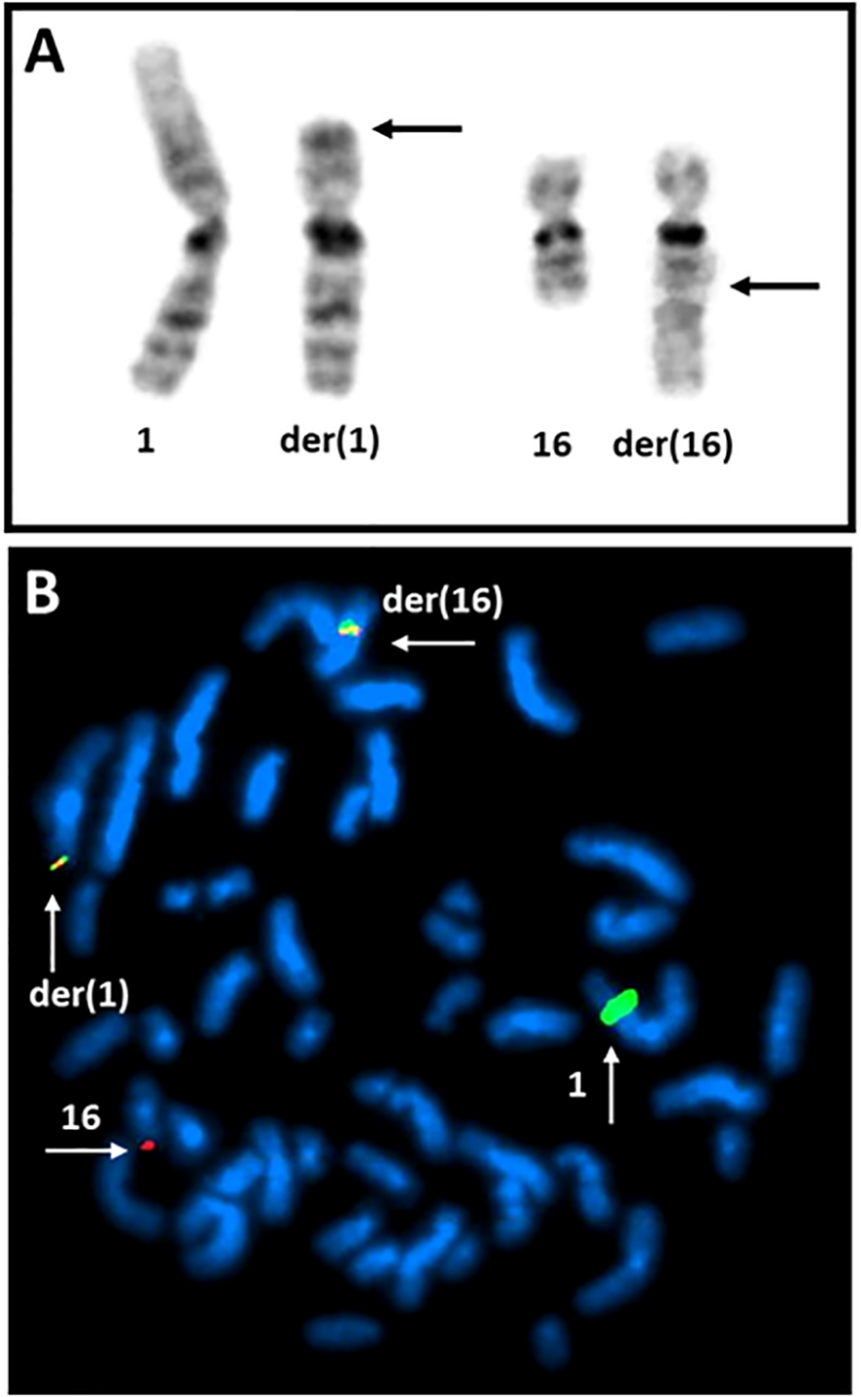
Detection of the 1;16-translocation by G-banding and FISH. **(A)** Partial karyotype showing the normal and derivative chromosome 1 and 16. Arrows are pointing to breakpoints. **(B)** FISH image showing the fusion between *NFIA* (mapping on 1p31 and labeled with green) and *CBFA2T3* (mapping on16q24 and labelled with red).

The G-banding analysis revealed a 49,XY,t(1;16)(p31;q24),+19,+2mar karyotype (**Figure 2**) in the samples from the bone marrow, lymph node, and pleural fluid.

FISH investigation showed no deletion for 5q, 7q, 9p, 20q, or the TP53-locus (17p13). No trisomy of chromosome 8 nor split of *KMT2A* (11q23), *IgH* (14q32), *E2A* (19p13), *MYC* (8q24), *PDGFRB* (5q), and *ABL2* (1q25) was seen. No fusion for *PML::RARA*, *RUNX1::ETO*, *CBFB::MYH11*, *BCR::ABL*, and *ETV6::RUNX1* was detected in the 200 nuclei analyzed. FISH using BAC probes mapping on the *NFIA* and *CBFA2T3* genes showed a fusion (yellow signal) on the derivative chromosomes 1 and 16 (**Figure 2**). aCGH did not show presence of genomic imbalances.

**Figure 3:**
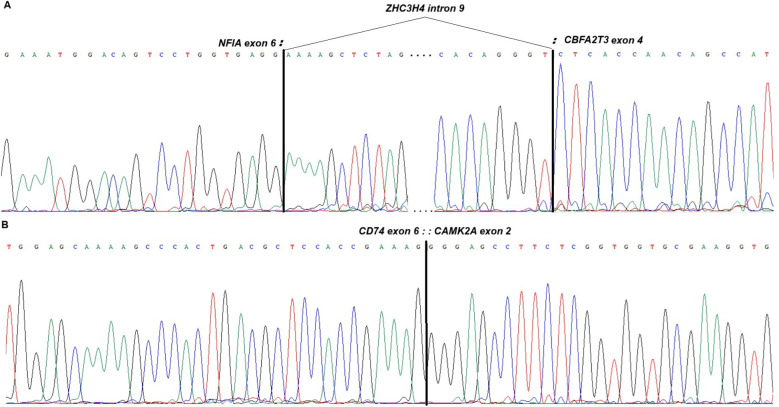
Partial Sanger sequencing chromatograms showing the fusion transcripts found in the erythrosarcoma. **(A)** Overview of *NFIA::CBFA2T3* chimera showing the insertion of the *ZHC3H4* between exon 6 of *NFIA* and exon 4 of *CBFA2T3*. **(B)** Overview of the *CD74::CAMK2A* transcript involving exon 6 and exon 2, respectively.

The *NFIA::CBFA2T3* fusion transcript, identified by FISH and confirmed by cycle sequencing, showed a fusion between exon 6 of *NFIA* (accession number NM_001134673.4) and exon 4 of *CBFA2T3* (NM_005187.6) (**Figure 3**). An insertion of a 216-base pair (bp) sequence from intron 9 of the gene *ZC3H4* on 19q13 (NM_015168.2) joined the chimera between exon 6 of *NFIA* and exon 4 of *CBFA2T3* (**Figure 3**).

A list of 27 transcripts was obtained from raw data of the sample after transcriptome sequencing (data not shown). No reads with an *NFIA::CBFA2T2* fusion were detected using the Fusion Catcher software. However, a specific transcript involving *CD74* and *CAMK2A*, spanning unique reads 10, was identified in the list. RT-PCR followed by direct cycling Sanger sequencing confirmed the presence of a fusion between exon 6 of the *CD74* gene (accession number NM_001025159.3) and exon 2 of the *CAMK2A* gene (accession number NM_015981.4; **Figure 3**).

The myeloid mutations panel detected a *KIT* Asp816His pathogenic variant (36%), which is typically associated with mastocytosis. Pathogenic variants on *FLT3* (ITD or TKD), *NPM1*, and *TP53* were not seen.

## 4 Discussion

Erythroid sarcoma (ES), also known as erythroblastic sarcoma, is a rare subtype of acute myeloid leukemia (AML) characterized by the proliferation of immature erythroid cells outside the bone marrow (1). It can occur as a primary tumor (*de novo*) or develop from a pre-existing MDS or MPN (4). The disease is challenging to diagnose and treat. It occurs in pediatric and adult patients, though it seems to evolve through different pathways. Children show simple karyotypes characterized by the presence of fusion genes, whereas adults have complex karyotypes and, in most cases, show the presence of pathogenic variants, with *TP53* being the most common alteration (13). A literature search for ES and AEL (including pure EL; PEL) showed a total of 23 cases of childhood disease, of which 20 were ES with involvement of extramedullary sites (5–14, 25–27), two from bone marrow only (17, 19), and one case described as sarcoma NOS (18) (**Supplementary Tables 1**, **2**). All cases but one have been cytogenetically and/or molecularly investigated. The present case is a new ES with a relatively simple karyotype, leading to a fusion transcript, which highlights the proposed genetic pathway of this disease in children. The *NFIA::CBFA2T3* fusion transcript has been reported to involve the fusion of *NFIA* exon 3, exon 4, or exon 6 with *CBFA2T3* exon 2, exon 3, or intron 1 (**Supplementary Table 1**). We report here, for the first time, a new breakpoint position involving exon 6 and exon 4, respectively, of the mentioned genes. Identification and reporting of novel breakpoint positions in fusion transcripts are fundamental, especially in a diagnostic context, where different molecular panels are designed with specific primer combinations and may therefore fail to detect transcript variants. Furthermore, the chimeric sequence contained an insertion of 216 bp from intron 9 of Zinc Finger CCCH-Type Containing 4 (*ZHC3H4*, mapping to 19q13), which introduced a shift in the sequence and a premature stop codon. This is the first time that an *NFIA::CBFA2T3* fusion is reported to have an insertion of a third sequence. The insertion of a sequence from a different gene could result from a more complex rearrangement than that seen at the chromosomal level. The presence of such an internal sequence was most likely the reason why neither the Fusion Catcher program nor the Fusion Plex, Pan-Heme panel (Archer) detected the original chimera.

The transcription factor NFIA is a key regulator of erythroid differentiation during early hematopoiesis (28). Similarly, CBFA2T3 (also known as ETO2) is a transcriptional corepressor that appears to play a role in erythroid differentiation through the coregulation of GATA1 targets (29). Molecular and functional studies have shown that *NFIA::CBFA2T3* initiates AEL/ES by suppressing gene expression programs associated with terminal erythroid differentiation and cooperates with *TP53* pathogenic variants to induce erythroleukemia (30). In the present case, no *TP53* pathogenetic variants were identified; therefore, the chimera must act in a slightly different manner with possible other genomic alterations, as it is hypothesized for the pediatric AEL/ES (13).

Further, a *CD74::CAMK2A* was also found in the present case. The chimera is between exon 6 of *CD74* and exon 2 of *CAMK2A*. Both genes are mapped to 5q32-q33.1, located approximately 110 kbp apart, and the transcript is in frame. The putative protein retains the MHC2-interacting domain from *CD74* and the catalytic domain of protein kinases from *CAMK2A*. The fusion has previously been identified in a B-ALL patient (31, 32). We believe the *NFIA::CBFA2T3* is the primary aberration in the present case, while the *CD74::CAMK2A* is a secondary one, both in a temporal and causal sense.

The WHO Classification of tumors (2024) recognizes acute erythroid leukemia, as a distinct entity under myeloid/erythroid neoplasms (33). ES is defined as a rare extramedullary tumor composed of erythroid precursors, typically occurring outside the bone marrow (e.g., soft tissue, lymph nodes, CNS) (5–11, 13, 14, 27). Although AEL and ES share similar morphological features, both consist of immature erythroid precursors, they differ in clinical presentation: AEL represents a systemic, marrow-based leukemia, whereas ES manifests as a localized, solid mass. The present case was a disseminated disease with minimal bone marrow involvement. Because ≥80% erythropoiesis with ≥30% proerythroblasts was not demonstrated in the bone marrow biopsy, the WHO diagnostic criteria for AEL were not fulfilled, and the neoplasm was therefore defined as a myeloid sarcoma. Since AEL and ES can overlap morphologically with other, clinically distinct myeloid malignancies such as AML with myelodysplasia-related changes, and MDS, it is fundamental to characterize them genetically. Furthermore, the genetic alterations appear to differ between pediatric and adult patients, i.e., simple versus complex karyotypes, with the presence of *TP53* pathogenic variants in the latter group (13).

Given the heterogeneity and rarity of the mentioned proliferations, it is challenging to make a precise diagnosis. However, since the presence of an *NFIA::CBFA2T3* and its variant or analogous fusions, *NFIA::RUNX1T1* and *ZMYND8::RELA*, has been identified in 14 of the pediatric cases (including the present report), we suggest that all these entities represent a single, distinct, molecularly defined subgroup of leukemias.

## Data Availability

The original contributions presented in the study are included in the article/**Supplementary Material**. Further inquiries can be directed to the corresponding author.

## Supplementary material

Supplementary Figure 1 Flowcytometric analysis of pleural effusion showing an immature CD117+ population, expressing the erythroid markers CD71, CD36 and CD235a, but was negative for CD105 and lineage markers cyCD79a, cyCD3 and cyMPO as well as all other myeloid and lymphoid markers tested.
