# Supplementary figures and images for: The NFIA::CBFA2T3 identifies a molecularly defined subgroup of acute erythroid leukemia/erythroid sarcoma
Source: Front Oncol. 2026 May 4;16:1809156. doi: 10.3389/fonc.2026.1809156 (PMC13181429; doi:10.3389/fonc.2026.1809156)

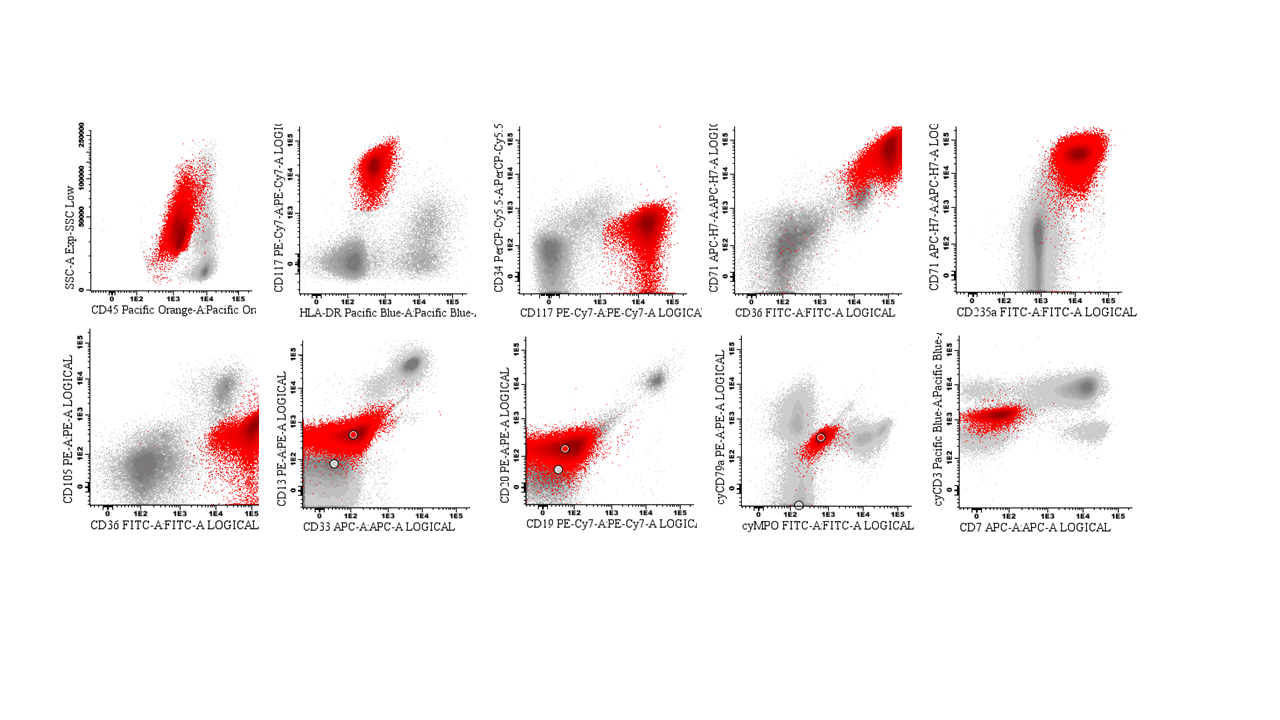

Supplement: Supplementary Figure 1 — Flowcytometric analysis of pleural effusion showing an immature CD117+ population, expressing the erythroid markers CD71, CD36 and CD235a, but was negative for CD105 and lineage markers cyCD79a, cyCD3 and cyMPO as well as all other myeloid and lymphoid markers tested. [file Image1.png]
