# Supplementary material for: The NFIA::CBFA2T3 identifies a molecularly defined subgroup of acute erythroid leukemia/erythroid sarcoma
Source: Front Oncol. 2026 May 4;16:1809156. doi: 10.3389/fonc.2026.1809156 (PMC13181429; doi:10.3389/fonc.2026.1809156)
Supplement: Supplementary file 2 [file Table1.docx]

**Supplementary Table_1. Overview of the clinical information, karyotypic findings, and molecular genetic data of pediatric patients with pure erythroid leukemia/erythroblastic sarcoma in the literature (including the present case).**

| **Case nr** | **Age at presentation** | **Sex** | **Diagnosis** | **Extramedullary disease/site** | **Bone marrow involvement** | **Karyotype** | **Fusion transcript with breakpoint position** | **Reference** |
| --- | --- | --- | --- | --- | --- | --- | --- | --- |
| 1 | 10 y | M | PEL | yes/spinal mass | -- | 47,XY,t(1;16)(p31;q2?2),del(7)(q31),+19,del(20)(p11) |  | [25] |
| 2 | 6 y | M | PEL | yes/abdominal wall | -- | 51,XY,t(1;16)(p31;q24),+6,+10,+15, +19,+21 |  | [26] |
| 3 | 3.5 mo | F | ES | yes/ovaries | yes | 47,XX,del(6)(q23q25),+7[21] |  | [6] |
| 4 | 15 mo | M | PEL | no | yes | 46,XY,der(1)t(1;1)(p31;q21),del(1) (p11p31),der(16)t(1;16)(p31;q24) [11] | *NFIA::CBFA2T3*  ex 3::ex 2 | [17] |
| 5 | 4 mo | M | EL | no | yes | 46,XY,t(11;20)(p11;q11)[8]/46,XY[2] | *ZMYND8::RELA*  ex 2::ex 2 | [19] |
| 6 | 3 mo | M | ES | yes/orbital mass | yes | 46,XY,del(6)(q25),add(19)(q13.3)[20] |  | [8] |
| 7 | 1 y 9 mo | F | PEL/MS | yes/periorbital | yes | 47,XX,t(11;20)(p15;p13),+add(17) (p13)[2]/47,sl,der(11)t(1;11)(q?21;q?24) [14]/47,sl1,add(8)(p23)[2] |  | [9] |
| 8a | 17 mo (S1) | F | PES | yes/orbital mass | no | 46,XX |  | [7] |
| 8b | 21 mo (S2) | F | PES | no/BM | yes | 46,XX,i(7)(q10),t(11;20)(q13;q11.2) |  | [7] |
| 9 | 2 y | F | PEL/PES | yes/CNS | no | 54,XX,+X,t(1;16)(p31;q24),+6,+7,+8,+8,+10,+14,+19[12]/55,sl,+15[8] | *NFIA::CBFA2T3*  ex 4::ex 2 | [5] |
| 10 | 3y | M | ES | yes/CNS | no |  | *NFIA::CBFA2T3* | [10] |
| 11 | 2 mo | F | PEL | yes/abdominal mass | yes | 46,XX,der(1)t(1;8)(p31.3;q21.3)t(1;2) (p13;p23),der(2)t(1;2)t(1;8),der(8) t(1;8)[3]/47,sl,+mar[8]/46,sdl1,−X[8]/ 46,XX[1] | *NFIA-RUNX1T1*  ex 3:: ex 2 | [12] |
| 12 | 6 mo | M | ES | yes/left humerus, intracranium, orbita, chest, CSF | no |  | *RCC1::LCK*  ex 2::ex 2 | [27] |
| 13 | 22 mo | F | ES | yes/left parotid region and CNS | no |  | *NFIA::CBFA2T3*  ex 6::ex 2 | [14] |
| 14 | <18 y |  | Sarcoma NOS | kidney | -- |  | *NFIA::CBFA2T3* | [18] |
| 15 | 3 y | M | ES | yes/CNS | -- |  | *NFIA::RUNTX1T1*  ex 2::ex 3 | [11] |
| 16 | 2 mo | M | AEL | yes/skull, skull base, liver, paraspinal and right shoulder soft tissue | yes |  | *CIC::NUTM2A* | [13] |
| 17 | 3 mo | F | AEL | yes/skull, with soft tissue extension; bilateral ovaries | yes | 47,XX,del(6)(q23q25),+7 |  | [13] |
| 18 | 6 mo | M | ES | yes/ascites, omentum | no |  | *NFIA::RUNX1T1* | [13] |
| 19 | 1 y | M | ES | yes/CSF, brain | no | t(1;16)(p31;q24),+6,+7,+8,+19 | *NFIA::CBFA2T3* | [13] |
| 20 | 2 y | F | AEL | yes/ retroperitoneal mass, bilateral inguinal LN | yes | 52,XX,der(1)t(1;1)(p32;q22q43),+6,+der(7)t(2;7) (q22;q32),+8,+15, der(16)t(1;16)(p13;q22),+19, +20, +r(21)(p11.2q22.1)[24]/97~104,XXXXX |  | [13] |
| 21 | 3 y | F | AEL | yes/right maxillary sinus, with extension into right pterygopalatine fossa and oral cavity | yes | 48,XX,+6,del(7)(p22p21),der(10)t(10;13)(q22;q12), t(11;20)(q13;q13.1),+19[14]/96, 47-50,X,add(X)(q26), del(1)(p32),+2,del(5)(q31),+7,-17,-21,+2-5 mar[cp2]idemx2[2] | *ZMYND8::RELA* | [13] |
| 22 | 6 y | F | ES | yes/abdominal soft tissue mass and LN | -- | 47-50,X,add(X)(q26),del(1)(p32),+2, del(5)(q31),+7,-17,-21,+2-5 mar[cp2] |  | [13] |
| 23 | 15 y | F | ES | yes/CSF, leptomeninges | no | 81,XX,-X,-X,der(1)t(1;8)(p?32;q?22.1)×2,+der(1)t(1:8),-2, -3,-3,-4,add(8)(q21)×2,+add(8) (q21),-9,t(10;16) (q22.1;p13.3)×3,-11,-12,-13,-14,-15,-(17)(q10),-18,-18, +19,-22,+mar[20] |  | [13] |
| 24a | 17 mo | M | ES | yes/colon (cells from BM) | No?  the WHO diagnostic criteria for a diagnosis of acute erythroid leukemia were not fulfilled in the bone marrow biopsies | 46,XY,t(1;16)(p31;q24),+19,+2mar[6]/46,XY[4] | *NFIA::CBFA2T3*  ex 6::ex 4 | Present case |
| 24b | 17 mo | M | ES | yes/colon (cells from LN) |  | 46,XY,t(1;16)(p31;q24),+19,+2mar[10] | NA | Present case |
| 24c | 17 mo | M | ES | yes/colon (cells from the pleural fluid) |  | 46,XY,t(1;16)(p31;q24),+19,+2mar[10] | *NFIA::CBFA2T3*  ex 6::ex 4 | Present case |

AEL= Acute Erythroid Leukemia, EL= Erythroid Leukemia, ES= Erythroid Sarcoma, MS= Myeloid Sarcoma, PEL= Pure Erythroid Leukemia, PES= Pure Erythrid Sarcoma

S1 = sample 1, S2= sample 2

BM = Bone Marrow, CNS = Central Nervous System, CSF = Cerebro spinal fluid, LN = lymph nodes

NOS = not otherwise specified, NA = not available
