# Supplementary material for: The NFIA::CBFA2T3 identifies a molecularly defined subgroup of acute erythroid leukemia/erythroid sarcoma
Source: Front Oncol. 2026 May 4;16:1809156. doi: 10.3389/fonc.2026.1809156 (PMC13181429; doi:10.3389/fonc.2026.1809156)
Supplement: Supplementary file 3 [file Table2.docx]

**Supplementary Table_2. Overview of the immunophenotype, treatment regimens, and clinical outcomes of pediatric patients with pure erythroid leukemia/erythroblastic sarcoma in the literature and the present case.**

| **Case nr** | **Immunophenotype** | **Treatment regimens** | **Clinical Outcome** | **Reference** |
| --- | --- | --- | --- | --- |
| 1 |  |  |  | [25] |
| 2 |  |  |  | [26] |
| 3 | CD71+,CD117+, glycophorin A +,CD36+,CD2+weak, CD64+,CD3-,CD79a-,CD16-,CD15-,CD33-,CD14-, CD38-,CD34-,CD123-,TdT-,CD45-,CD1a-,CD4-,CD8-, CD5-,CD7-,CD8-,CD10-,CD11b-,CD19-,CD45RO-, CD61-hemoglobin A + | Pediatric Oncology Group Protocol 9317 with etoposide/ifosfamide | CR | [6] |
| 4 |  | NOPHO-AML 2004 protocol | Died of resistant  disease 5 months after diagnosis | [17] |
| 5 | CD45-,CD71+,glycophorin+,CD36+,CD13-,CD33-, CD11b- | HLH-protocol followed by transplantation | CR | [19] |
| 6 | CD99+,CD71+,CD43+(focally),CD45-,CD34-,Tdt-, PGP9.5-,synaptophysin-,CD68-KP1-,hemoglobinA-, Fli-1-,E-cadherin- | Chemotherapy with alternating cycles of ifosfamide and etoposide, and cyclophosphamide, vincristine, and doxorubicine | Died 8 weeks after initial presentation | [8] |
| 7 | CD45-,CD34-,CD117-, CD42b-,CD61- | BFM AML high risk protocol | Morphological remission in consolidation phase | [9] |
| 8a | CD36+weak,CD45+,CD105a+,CD71+,CD117+ weak, CD34+ weak,CD33+ weak,HLADR+, glycophorin A intense | Second line chemotherapy | No response, died of progression | [7] |
| 8b | CD36++,CD45+weak,CD105a+,CD71+,CD117+weak, CD34+ weak,CD33+ weak |  |  | [7] |
| 9 | aberrant cell population (90%),CD36bright,CD71+, CD117 heterogeneous,glycophorin A heterogeneous, CD14-,CD15-,CD34-,CD38-,CD45-,CD64-,E-cadherin+, CD71+,hemoglobin + (variable) | Initial induction chemotherapy AAML1031, followed by induction II AAML1031-high risk, bone marrow transplant after six months, followed by additional IT chemotherapy and radiation | CR | [5] |
| 10 | CD43+,CD117+,c-MYC+,CD34-,MPO-,lysozyme-, CD61-,CD3-,CD20-,PAX-5-, CD10-,CD30-,TdT-,Ki-67 (95%),CD71+,E-cadherin+,glycophorin A+ | Chemotherapy (not otherwise specified) | Improvement (at time of writing the article) | [10] |
| 11 | CD71+,CD117+,CD43+ (focal),E-cadherin +,epithelial membrane antigen+,hemoglobin A+,CD13-,CD33-, CD34-,CD41-,CD45-,CD61-,CD117-,cMPO-, cCD3/sCD3-,cC79a-,CD38-,CD15-,CD19-,HLA-DR-,  CD123-,CD14-,CD64-,CD4-,CD16-,CD2-,CD5-,CD7-, CD56-,CD20-,CD10- | Vincristine, doxorubicine, and cyclophosphamide, followed by induction therapy per  Children’s Oncology Group protocol AAML1031 | CR, then relapse | [12] |
| 12 | CD1a-,CD4-,CD8-,CD13-,CD14-,CD19-,CD20-,CD25-, CD33-,CD34-,CD45-,CD56-,CD117-,CD36+,GPA+,  E-cadherin+ | Multiagent chemotherapy with cytarabine, anthracyclines, and etoposide, including intrathecal injection of cytarabine, methothrexate, and hydrocortisone followed by allogenic cord blood transplantation | CR | [27] |
| 13 | CD43+,CD99+,CD45-,CD30-,CD34-,TdT-,CD1a-, glycophorin-,E-cadherin-,MPO-,CD15-,CD33-, lysozyme-,CD42B-,CD61-,CD19-,CD20-,CD79a-, PAX5-,BCL2-,BCL6-,CD3-,CD2-,CD5-,CD7- | Intravenal chemiotherapy with dexamethasone, cytarabine,cyclophosphoamide, and intrathecal methotrexate | Clinical improvement and decreased enhancement and size of the lesion | [14] |
| 14 | -- | -- | -- | [18] |
| 15 | CD117+,CD43+,E-cadherin-,CD34-,CD45-,CD3-,CD20- | First line vincristin, doxorubicin, cyclophosphamide, etoposide and ifosphamide, followed by Myechild 01Trial with mitoxantrone and cytarabine | Died three months after initial presentation | [11] |
| 16 | ALK-,CD1a-,CD2-,CD3-,CD4-,CD5-,CD7-,CD8-, CD10-,CD30-,CD56-,TdT-,CD20-,CD79a-,CD13subset, CD61-,CD99-,CD163- | COG AAML1831 | CR, then relapse | [13] |
| 17 | CD1a-,CD2dim,CD3-,CD4-,CD5-,CD7-,CD8-,CD10-, CD56subset,TdT-,CD19-,CD79a-,CD11b-, CD13subset,CD14-,CD15-,CD33-,CD38-,CD61-, CD64dim,CD68-,D123- | Pediatric Oncology Group protocol 9317 (fractionated cyclophosphamide, vincristine, and doxorubicin), then platinum-based regimes (course #2) with cyclophosphamide, carboplatin, and VP-16, followed by another course of Protocol 9317, followed by stem cell transplant | CR | [13] |
| 18 | CD3-,CD30 30%,TdT-,CD20-,CD61- | AAML1831; cytarabine×10 days; daunorubicin day 1,3,5 |  | [13] |
| 19 | CD3-,CD4-,CD5-,CD7-,CD8-,CD10-,CD22-,CD79a-, CD11b-,CD11c-,CD14-,CD15-,CD33-,CD38-,CD41-, CD42-, CD61-,CD64- | Idarubicin, cytarabine, venetoclax; azacitidine, venetoclax, medium-dose cytarabine; azacitidine, Idarubicin, venetoclax; Azacitidine, venetoclax, cladribine | CR | [13] |
| 20 | ALK-,CD2-,CD3-,CD4-,CD56-,TdT-,CD20-,CD61-, CD99- | NBL regimen (vincristine, dexrazoxane, doxorubicin, and cyclophosphamide) | CR | [13] |
| 21 | CD3- | -- | -- | [13] |
| 22 | ALK-,CD3-,CD4-,CD5-,CD7-,CD8-,CD30-,CD56-,TdT-, CD20-,CD138-,CD33-, CD42-,CD61-,CD68-,CD99-, CD123- | -- | -- | [13] |
| 23 | ALK-,CD2-,CD7+,CD30-,CD56-,CD138-,CD11b-, CD25-,CD33-,CD41-,CD123- | -- | -- | [13] |
| 24 | CD43+ (strong),CD117+ (strong),BRG+, E-cadherin+, INI-1+,CD99-/+,p53-/(+),CD56-(+few cells),c-myc+, CD2-,CD3-,CD4-,CD7-,CD14-,CD20-,CD25-,CD34-, CD30-,CD45-,CD61-,CD68KP1-,CD163-,AFP-,ALK-, Desmin-,ERG-,Factor 8-,GATA3-,Glycophorin-,Inhibin-, Lysozyme-,MPO-,MUM1-,MyoD1-,Myogenin-,Oct3/4-, Pax5-,PHOX2B-,PLAP-,SALL4-, Synaptophysin-,TdT-, WT1-,EBV(ISH)-, S100- | NOPHO AML 2012 protocol (with etoposide monotherapy first and then courses with MEC, ADE, and FLA), followed by allogenic stem cell transplantation | CR | Present case |
